# The Common Mosquito (*Culex pipiens*) Does Not Seem to Be a Competent Vector for Hepatitis E Virus Genotype 3

**DOI:** 10.3389/fvets.2022.874030

**Published:** 2022-04-26

**Authors:** Mario Frías, Laia Casades-Martí, María Á. Risalde, Pedro López-López, Raúl Cuadrado-Matías, Antonio Rivero-Juárez, Antonio Rivero, Francisco Ruiz-Fons

**Affiliations:** ^1^Virología Clínica y Zoonosis, Instituto Maimónides de Investigación Biomédica de Córdoba (IMIBIC), Hospital Universitario Reina Sofía de Córdoba, Universidad de Córdoba, Córdoba, Spain; ^2^CIBERINFEC, ISCIII – CIBER de Enfermedades Infecciosas, Instituto de Salud Carlos III, Madrid, Spain; ^3^Grupo Sanidad y Biotecnología, Instituto de Investigación en Recursos Cinegéticos, IREC (CSIC-UCLM-JCCM), Ciudad Real, Spain; ^4^Grupo de Investigación en Sanidad Animal y Zoonosis, Departamento de Anatomía y Anatomía Patología Comparada y Toxicología, Facultad de Veterinaria, Universidad de Córdoba, Córdoba, Spain

**Keywords:** vector competence, HEV, mosquito, *Culex*, experimental infection

## Abstract

An experimental infection approach was used to estimate the competence of the common mosquito, *Culex pipiens*, for hepatitis E virus replication and transmission, using an isolate of hepatitis E virus genotype 3 of human origin in varying infectious doses. The experimental approach was carried out in biosafety level 2 conditions on three batches of 120 *Cx. pipiens* females, each using an artificial feeding system containing the virus in aliquots of fresh avian blood. Mosquitoes from each batch were collected 1, 7, 14, and 21 days post-infection (dpi) and dissected. The proboscis was subjected to forced excretion of saliva to estimate potential virus transmission. HEV RNA presence in abdomen, thorax, and saliva samples was analyzed by PCR at the selected post-infection times. HEV RNA was detected in the abdomens of *Cx. pipiens* females collected 1 dpi in the two experimentally-infected batches, but not in the saliva or thorax. None of the samples collected 7–21 dpi were positive. Our results show that *Cx. pipiens* is not a competent vector for HEV, at least for zoonotic genotype 3.

## Introduction

Hepatitis E virus (HEV) is the leading cause of acute viral human hepatitis worldwide, with significant morbidity and mortality rates in both developed and developing countries (1–3). In developed countries, the main route of transmission of HEV genotypes 3 and 4 (4) is from animal reservoirs to humans (1). These zoonotic genotypes have been detected in a wide variety of mammals, with swine being considered the main host (5) and consequently consumption of pork products the main transmission route (6). Among other mammal species that are hosts for zoonotic HEV genotypes, wild cervids represent other zoonotic source of infection (7, 8).

While the main transmission routes have been established, it has been suggested that there is a seasonal pattern in both human and animal populations, although it is not clear why. With respect to this, our group described a seasonal pattern of HEV infection in Spain, in which the number of wild boar with active infection decreased in the colder months (9). Likewise, a study carried out in the southwest of England found that the numbers of diagnosed cases of HEV in humans peaked in the spring and summer months (10). There is currently no explanation for this pattern, although it suggests an unknown transmission route with strong seasonal modulation. A plausible hypothesis is the involvement of an arthropod vector with high spring-summer activity (11, 12). Two main arthropod vectors, ticks, previously suggested by our group (13), and mosquitoes, which have not yet been evaluated, could be linked to HEV maintenance and transmission due to their intimate ecological association with the wild ungulates that maintain zoonotic HEV genotypes, as well as their wide distribution, and abundance (14).

Due to human behavioral ecology and globalization, the prevalence and spatial distribution of mosquito-borne pathogens have increased dramatically in endemic countries. Several mosquito-borne pathogens have also spread to new regions of the world (15, 16). Mosquitoes of the *Culex pipiens complex* are the most widespread and abundant in Europe and are currently responsible for the emergence of several relevant mosquito-borne viruses in this region, such as West Nile virus and Usutu virus (17). As a result, several studies have evaluated the possible role of this species in the transmission of viruses other than arboviruses (18–20). In connection with this, the *Culex pipiens* complex includes five mosquito species, including the nominal species *Cx. pipiens*, which has two different biotypes, *Cx. pipiens pipiens* and *Cx. pipiens molestus* (21, 22), and hybridisations between the two biotypes have also been described (23, 24). To estimate the vector competence of mosquitos for pathogens, it is essential to set up controlled artificial colonies in the laboratory capable of generating the multitude of specimens of the target species required for experimental study, and also to develop and implement efficient blood-feeding tools and protocols, which are also necessary to simulate natural infection during experimental trials. Using such experimental models, it has been possible to demonstrate the vectorial competence of certain species of mosquitoes for pathogens such as West Nile virus or Zika virus (25, 26). Based on the seasonal patterns of infection observed, experimental infection studies are needed to evaluate the possibility of HEV replication in the salivary glands of mosquitoes after feeding on HEV-infected hosts which would then be transmitted through mosquito bites. The main objective of this study was to test the hypothesis that the common mosquito is competent to transmit zoonotic HEV genotypes.

## Materials and Methods

### Culex Pipiens Colony

Throughout March 2020, mosquito larvae were collected from a rainwater collection pit (38.99417, −3.925073) situated in the Spanish Game and Wildlife Research Institute (IREC) in Ciudad Real, south-central Spain. Larvae were taken to the entomology laboratory, morphologically identified to species level using specific taxonomic keys (21), then sorted by species and kept in containers in deionized water supplemented with sinking pellet fish food until pupation (Vipagran, Sera, Germany). The mosquitoes were monitored daily to collect the newly emerged pupae, which were transferred to hatching containers (model G861, Entomopraxis, Spain). The emergence of adult mosquitoes from the pupae was monitored daily; adults were then transferred to BugDorm insect cages (model G4E2222, Entomopraxis, Spain). The colony was started with 250 adult *Cx. pipiens* from the F1 generation, which were kept at constant room temperature (21 ± 3°C), with a 12/12 h light/dark cycle, and constant relative humidity of 85 ± 5%. The molecular identification and characterization of colony mosquitoes was performed by targeting the CQ11 microsatellite locus using a qPCR assay as described elsewhere (27). Molecular analysis of adult individuals collected from the F1 generation confirmed the results of morphological identification.

**Figure 1:**
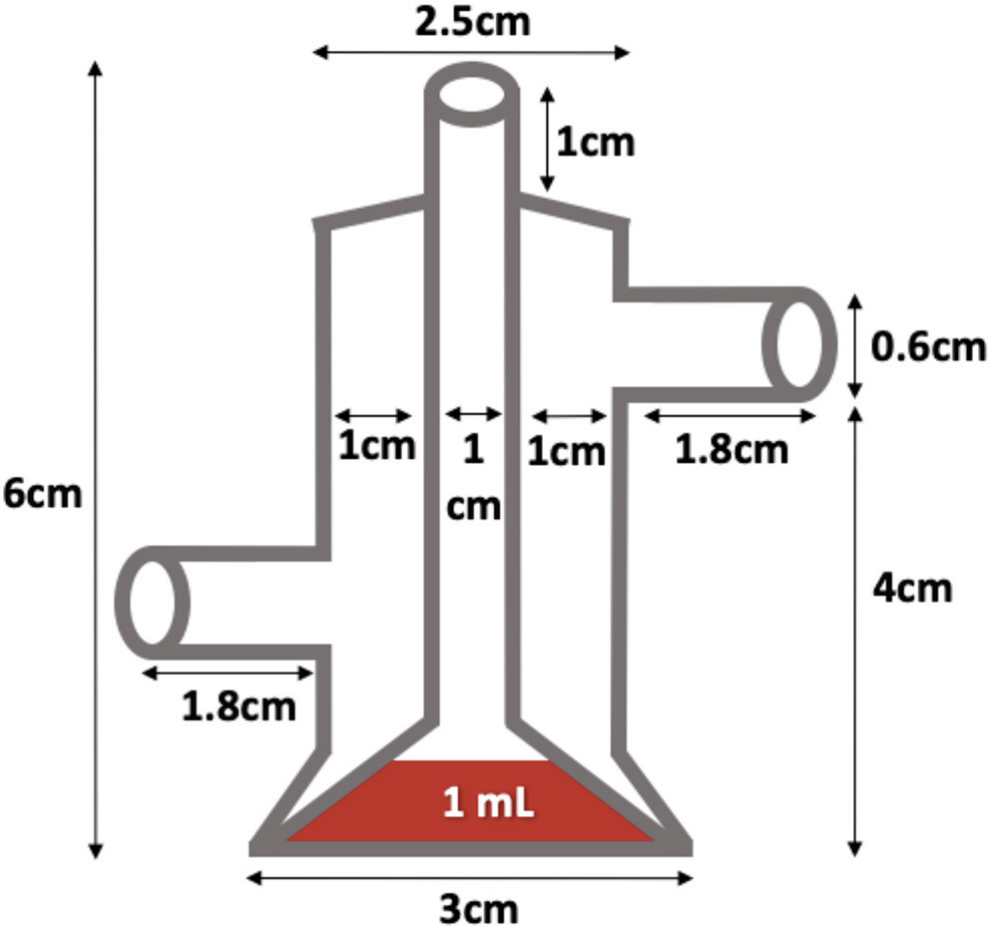
Homemade feeder used in this study.

**Figure 2:**
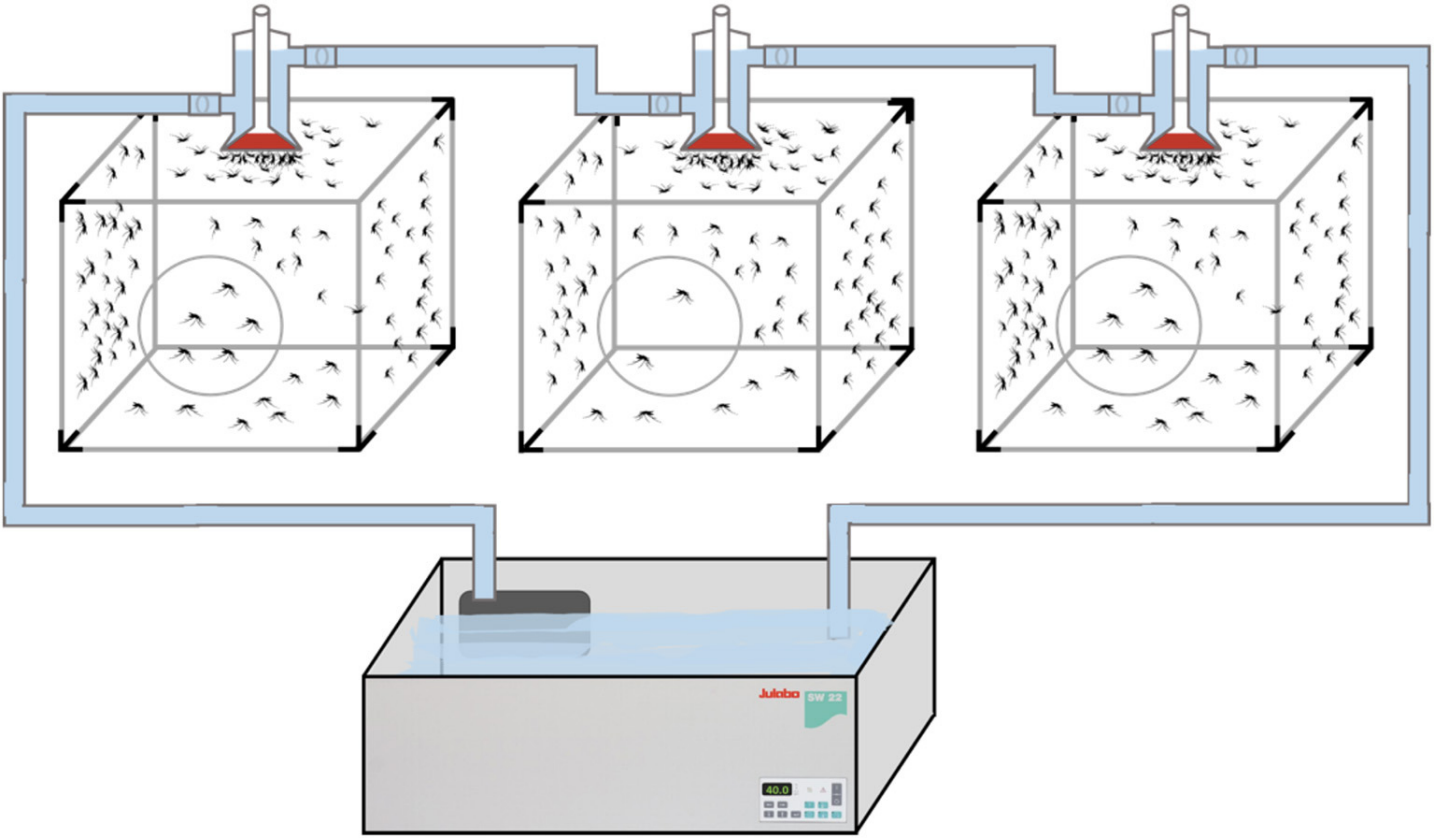
Experimental feeding set-up with constant flow of circulating water.

**Figure 3:**
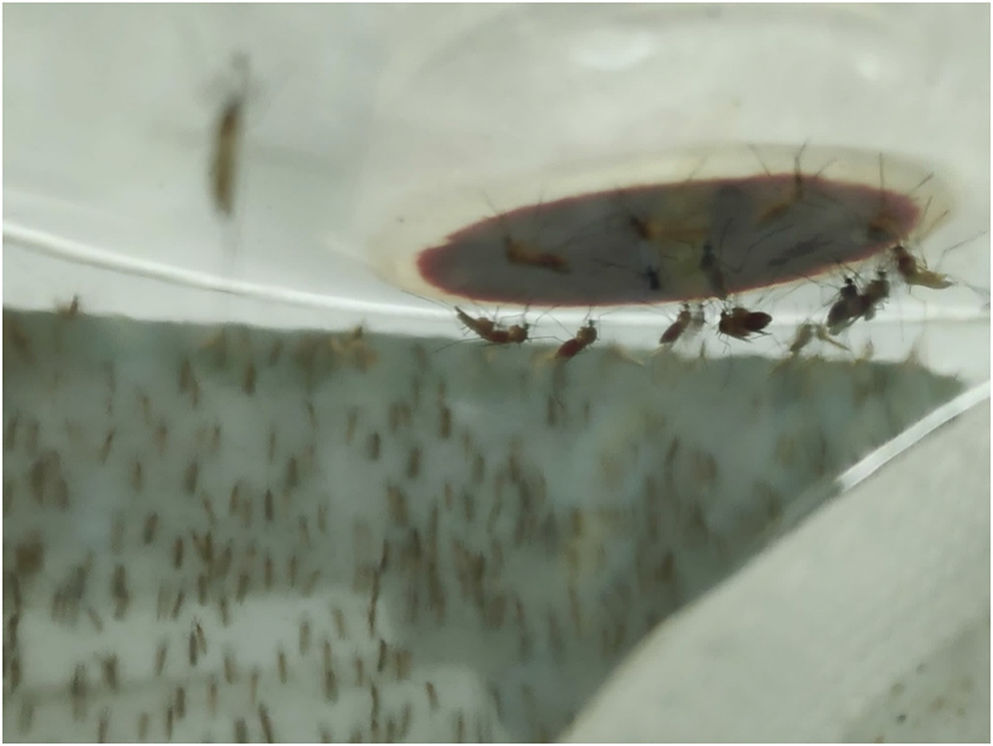
Base of the feeder during experimental infection.

The adult mosquitoes were fed using filter paper immersed in a container of water containing 3 g of pasteurized honey. In parallel, a small container with deionized water, lightly supplemented with sinking pellet food, was placed in every cage containing egg-laying adults. Batches of eggs were collected daily and transferred to plastic trays for hatching and larval development. Every 2 weeks, adult female mosquitoes were fed with fresh poultry blood collected from a local abattoir. Blood was collected from chickens in 500 mL containers coated with lithium-heparin at a concentration of 20 IU/mL. Two aliquots per blood batch were analyzed to rule out the presence of avian HEV prior to mosquito feeding and prevent potential interference with the experiment. A customized glass feeder made by a glass-artisan was used to dispense blood to the mosquitoes (Figure 1). Its design was based on one described by Rutledge et al. (28), which had a base that allowed ~40 mosquitoes to feed at once and a sealed water circulation system that provided a continuous flow of water at 41°C to keep the blood warm and optimize the feeding rate (Figure 2). To simulate the skin of an avian host, the base of the feeder was covered with quail skin (commercially farmed quails) obtained from a local abattoir (Figure 3). The blood was pre-warmed to 41°C in a water bath (model SW22, Julabo, Germany), which was also employed as a hot water source to keep the blood warm inside the device. The circulation of water between water bath and device and vice versa was achieved with a small water pump (CompactON 300, Ehein, Germany) connected to the feeding device by rubber hoses.

### HEV Inoculum

HEV replication in cell culture is extremely difficult to achieve and current models are poorly standardized (29, 30). As in other studies (31, 32), we used serum from viremic human patients for the experiment. Two viable infectious HEV inocula were available, consisting of two samples of serum from patients diagnosed with acute HEV infection with a zoonotic genotype. One milliliter of each serum sample was stored at −80°C until the day of the experimental trial. Viral loads (VL) estimated from C_t_ and standard curve performed with a known VL, were 16,660,319 IU/mL for serum no. 1 (S_1_), and 4,992,494 IU/mL for serum no. 2 (S_2_). The HEV RNA sequences of both samples, belonging to a fragment of the ORF2 region of the virus, were registered in GenBank with accession numbers MN628566 and MN628567. This sequencing allowed both strains to be designated genotype 3f, which is one of the dominant zoonotic genotypes in Europe.

### Experimental Approach

The experiment was designed to simulate real infection of *Cx. pipiens* mosquitoes feeding on viremic hosts in order to estimate their ability to become infected and transmit HEV. This was another reason for choosing samples from proven clinically infected viremic hosts with HEV for the experiment. Since there were no previous trials to estimate the potential competence of mosquitoes for transmitting HEV, we designed the experiment as a single-vector competence study with three mosquito batches: (i) B_1_, which were fed with avian blood containing S_1_ with a final infectious dose of 8,330,160 IU/mL; (ii) B_2_, fed with avian blood containing S_2_ with a final infectious dose of 2,496,247 IU/mL; and (iii) B_3_, a control batch in which mosquitoes were fed with non-HEV-infected avian blood. Each batch initially contained 120 14–21-day-old adult *Cx. pipiens* females from the F7 and F8 generations. We selected 14–21-day-old females after observing that the highest proportion of *Cx. pipiens* females in our colony that fed successfully on the artificial feeding device was of this age. Likewise, to optimize feeding, the sugared food was withdrawn 24 h before the experimental trial.

The experimental infections were carried out in a BSL2 vertical laminar flow cabinet (Mars Pro 1200, LaboGene, Denmark) in a BSL2 laboratory suite. The laminar air flow was reduced to 0.25 m/s to avoid disturbing the mosquitoes in flight, reduce the drying potential of the airflow and the resulting dehydration stress in the mosquitoes, which might impair feeding and survival. The experiment was timed to coincide with the preferred feeding time of mosquitoes. We had previously observed that the proportion of females that successfully fed on blood was highest at dusk. The laboratory lights were turned off and only the BSL2 booth light was kept on (50 lux) to simulate twilight before the mosquitoes were brought in from the entomology lab.

Blood inocula containing infectious HEV were freshly prepared immediately prior to feeding them to the mosquitoes in a BLS2 cabinet adjacent to the one designated for the cages containing mosquitoes. Deep-frozen aliquots of S_1_ and S_2_ were brought to room temperature (RT). Five hundred microliters of each aliquot were smoothly mixed with 500 μL of chicken blood that had previously been brought to RT from 4°C in labeled, sterile 2 mL tubes. For B_3_, 500 μL of blood were mixed with the same volume of sterile PBS. The three tubes were brought to 41°C in a water bath, from where they were administered to the sterile, glass feeding devices. A different artificial feeding device was employed for each batch. The blood inoculum was kept in the device for up to 30 min to allow the maximum number of *Cx. pipiens* females to feed. The three batches were fed at the same time, not sequentially. Chicken blood was collected from a local abattoir early on the same day of the experiment and the presence of avian HEV was ruled out. After feeding, non-engorged females were removed from each batch with hand-held mosquito aspirators. The three batches of mosquitoes were kept in the BSL2 lab for 21 days after infection for sequential sampling.

### Mosquito Dissection and RNA Extraction

**Figure 4:**
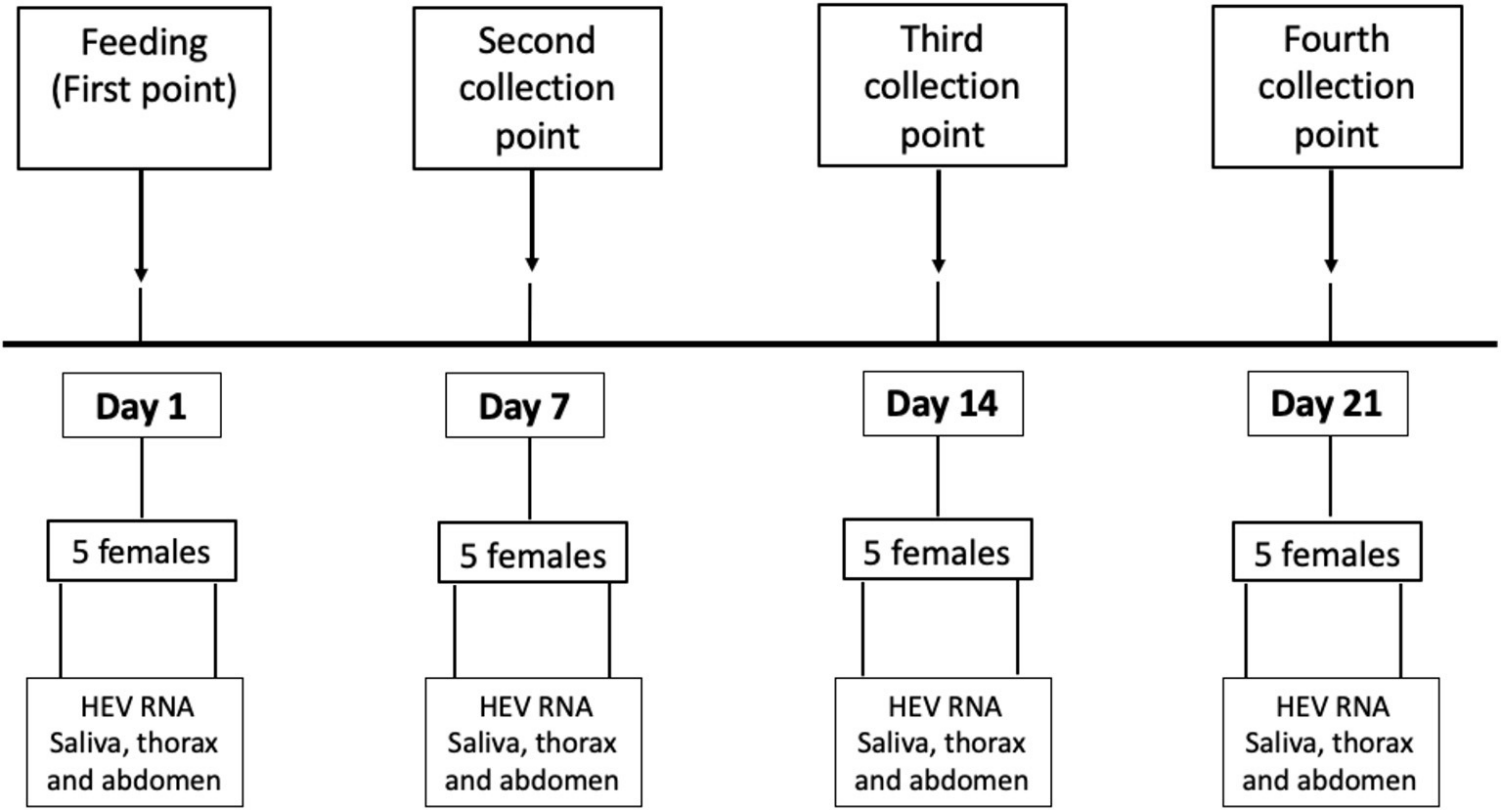
Methodological and chronological design of the study.

Mosquito vector competence was evaluated by determining the presence of HEV RNA by RT-PCR in different parts of the mosquito anatomy at 4 time points after feeding: day 1 (1 dpi), day 7 (7 dpi), day 14 (14 dpi), and day 21 (21 dpi). Figure 4 shows the chronology and methodological approach of the experiment. The blood inoculum remaining in each device after feeding the mosquitoes was collected in 1.5 mL sterile tubes and the three samples were deep frozen at −80°C.

At each selected time point, five mosquitoes were randomly removed from each batch separately and processed for molecular detection of HEV RNA. The mosquitoes were first immobilized by cold shock for 5 min. The legs and wings were then removed using the bevel of a 25G disposable needle and stored together in a 1.5 mL sterile nuclease-free tube. The head, thorax, and abdomen of each mosquito were dissected and placed separately into 1.5 mL tubes for RNA purification. To collect mosquito saliva as the best indicator of virus transmission potential, a forced salivation extraction technique was used, as described elsewhere (33). Briefly, the proboscis of each mosquito was dissected from the mosquito head and inserted into a 10 μL sterile pipette tip filled with 5 μL of fetal bovine serum (FBS) for 45 min. After that, the proboscis was removed and the FBS was kept frozen at −80°C in 200 μL sterile and nuclease-free tubes. The rest of the samples from each mosquito were also kept frozen at −80°C. All dissections were carried out in a BSL2 vertical flow cabinet, using disposable and/or sterile tools to avoid cross-contamination between different parts of the same mosquito, and also between different mosquitoes and batches.

The NucleoSpin RNA plus kit (Macherey-Nagel, Düren, Germany) was used for RNA isolation from the FBS containing mosquito saliva, the thorax where the salivary glands are located, and the abdomen where the blood meal is processed in the mosquito gut.

### HEV RNA Detection

For detection of HEV RNA, a nested RT-PCR was used, designed to amplify a fragment of the ORF3 region of the virus. For the PCR reaction, the Access RT-PCR system (Promega Corporation, Madison, USA) was used. The outer and inner primers (20 μM) employed and thermal profile were as described by Inoue et al. (34). A World Health Organization Standard HEV strain, supplied by the Paul-Ehrlich-Institute (code 6329/10), was used as positive control. Chicken blood was also tested to confirm the absence of avian Hepatitis E virus (*Orthohepevirus* B). For this, the RNA of 200 μL of whole heparinized blood per aliquot was purified using TRI reagent BD (Sigma-Aldrich, St. Louis, USA), following the protocol provided by the manufacturer. RNA samples were immediately analyzed with a RT-PCR system protocol able to detect all *Orthohepevirus* species (35). Sample cross-contamination during nucleotide extraction and PCR was excluded by including a negative control (nuclease-free water; Promega, Madison, WI, USA) in every 10 purified/PCR samples that were all tested by PCR.

## Results and Discussion

DNA barcoding of F1 generation mosquitoes confirmed the results of the morphological classification as *Cx. pipiens*. Identification of *Cx. pipiens* biotypes in the F7 generation found a mixture of biotypes, including *Cx. pipiens* biotype *pipiens, Cx. pipiens* biotype *molestus*, and hybrids of the two biotypes. The presence of hybrid *pipiens/molestus* mosquitoes has been described previously (23, 24, 36); indeed, it has also been reported that formation of *Culex* spp. hybrids is common in colonies with several different species (37).

None of the blood samples collected from the abattoir were positive for HEV RNA, including the one collected for the experiment, nor were they positive for avian HEV (*Orthohepevirus* B). In the experimental feeding study, 99, 91, and 87 engorged mosquito females were obtained from B_1_, B_2_, and B_3_ batches, respectively, giving a feeding rate of 82.5, 75.8, and 72.5%. At 1 dpi, five mosquitoes were randomly selected from each batch and the presence of HEV RNA was detected in all abdomen samples collected from the female mosquitoes fed in batches B_1_ and B_2_, thus confirming that the virus was actually ingested with the bloodmeal. All the females collected from the control group (B_3_) at 1 dpi were PCR-negative for HEV RNA. Virus RNA was not detected in other mosquito samples (saliva and thorax) at 1 dpi. The presence of HEV RNA at 1 dpi and only in the abdomens of *Cx. pipiens* females that fed on infected blood confirms that the virus was effectively ingested with the bloodmeal and that mosquito exposure to the virus was successful.

In both the experimental and the control batches, none of the samples collected at 7, 14, and 21 dpi were positive for HEV RNA. Table 1 shows the results per batch at each collection time point. No other sections of the mosquito anatomy were positive at 1 dpi in the experimentally infected batches, showing that there was no early viral replication in the mosquitoes. The negative findings at 7–21 dpi in each sample taken from the experimentally-infected mosquitoes shows that the virus and even RNA is efficiently degraded in the mosquito midgut, and demonstrates the lack of competence of *Cx. pipiens* mosquitoes for the replication, maintenance, and transmission of HEV genotypes of zoonotic origin. The vector competence of *Culex* spp. shown for certain arboviruses, such as the West Nile virus, lies in the ability of these viruses to overcome various tissue barriers in the mosquito organism (38). In this study, the absence of HEV in all mosquitoes tested on and after day 7 post-inoculation suggests that the virus is unable to overcome midgut barriers and that *Cx. pipiens* mosquitoes are refractory to infection, preventing virus spread to the salivary glands, and further transmission.

**Table 1:** Number of samples positive for HEV RNA at each point.

	**Day 1**			**Day 7**			**Day 14**			**Day 21**		
	**Saliva**	**Thorax**	**Abdomen**	**Saliva**	**Thorax**	**Abdomen**	**Saliva**	**Thorax**	**Abdomen**	**Saliva**	**Thorax**	**Abdomen**
Experimental batch (B_1_)^a^	0/5	0/5	5/5	0/5	0/5	0/5	0/5	0/5	0/5	0/5	0/5	0/5
Experimental batch (B_2_)^b^	0/5	0/5	5/5	0/5	0/5	0/5	0/5	0/5	0/5	0/5	0/5	0/5
Control Batch (B_3_)	0/5	0/5	0/5	0/5	0/5	0/5	0/5	0/5	0/5	0/5	0/5	0/5

a *Inoculum used with 16,660,319 UI/mL.*

b *Inoculum used with 4,992,494 UI/mL*.

While this is the first study to evaluate the vector competence of mosquitoes for HEV transmission, it is not the first time that the vector capacity of *Culex* spp. for hepatotropic viruses has been assessed. The study carried out by Chang et al. (20) analyzed the vector competence of *Culex quinquefasciatus* (a member of the *Cx. pipiens* group) for the transmission of hepatitis C virus (HCV). As in this study, Chang et al. found the presence of the virus in the first few days after feeding HCV-infected blood to *C. quinquefasciatus*. However, they did not find evidence of virus replication and the rate of HCV RNA detection declined quickly. Likewise, another study was carried out to test the persistence of hepatitis B virus in a colony of *C. quinquefasciatus* (39). The authors did not detect the presence of the virus in salivary glands and concluded that the probability of transmission of hepatitis B virus in this species was low. All this suggests that hepatotropic viruses, regardless of the viral family, are not transmitted by this route.

Even though HEV has a wide host range and is maintained efficiently by wild ungulates (7, 9), only two studies have evaluated the presence of the virus in arthropods. In a previous study, we found that ticks (*Hyalomma lusitanicum*) that had fed on viremic animals were positive for HEV, although it was not possible to demonstrate the vectorial capacity of these arthropods (13). In another study, Vandeweyer et al. (40) assessed the risk of transmission of certain foodborne pathogens by eating insects and did not find HEV RNA in 92 crickets (*Acheta domesticus*) analyzed. The authors concluded that the risk for human consumption from eating these arthropods was low.

We focused on studying the competence of *Cx. pipiens* mosquitoes as it is the most widespread and abundant species, but the negative results obtained still leave room to hypothesize that other mosquito species could play a role in HEV transmission, since we could not rule this out. We also focused on a zoonotic HEV genotype that is shared by humans and animals and cannot rule out that other genotypes replicate more effectively in *Cx. pipiens* and other mosquitoes. However, the rapid clearance of virus in the *Cx. pipiens* midgut in less than a week suggests that this species may be refractory to infection by other HEV genotypes.

We set out to simulate the natural conditions in which HEV transmission might occur at the host-mosquito interaction interface by selecting two sera from patients with confirmed acute HEV infection and high virus titers with a very low probability of loss of infectious potential and virulence. However, we could not confirm virus infectivity and viability due to the difficulties of growing the virus on conventional cell lines, as has also been reported previously (30). We are however confident that the protocol employed to preserve infectious patient sera until the experiment and the short period of time between collection and experiment maintained the infectious potential of the virus in the samples.

In conclusion, this study adds to the knowledge on HEV transmission between hosts. *Culex pipiens* does not seem to be a competent vector for zoonotic HEV genotype 3. This leaves the reason for the observed seasonality of HEV infection incidence unexplained, which means that it is necessary to evaluate other possible transmission routes that would explain this behavioral pattern of HEV.

## Data Availability Statement

The raw data supporting the conclusions of this article will be made available by the authors, without undue reservation.

## Ethics Statement

The sera inoculated belong to patients who signed an informed consent form. The activities carried out with the mosquitoes were conducted according to ECDC guidelines for the surveillance of invasive mosquitoes in Europe (https://www.ecdc.europa.eu/sites/default/files/media/en/publications/Publications/TER-Mosquito-surveillance-guidelines.pdf).

## Author Contributions

FR-F, AR-J, MF, and LC-M: concept and design. LC-M, MF, RC-M, and FR-F: mosquito colony maintenance. MF, LC-M, and MR: RNA extraction and HEV RT-PCR. MF, LC-M, AR-J, AR, and FR-F: draft the manuscript. AR-J, AR, MF, and FR-F: funding. All authors critical revision of the manuscript, contributed to the article, and approved the submitted version.

## Funding

This study was supported by Projects GCL2017-89866-R and E-RTA2015-0002-C02-02 funded by Spanish Ministry for the Science and Innovation (MCI), SBPLY/19/180501/000321 funded by Regional Government of Castilla-La Mancha and the European Social Fund (ESF) and PIN-0477/2017 funded by Fundación Progreso y Salud, Consejería de Salud de la Junta de Andalucía. RC-M and LC-M acknowledges funding by MCIU, ESF, and the University of Castilla-La Mancha through Contract PRE2018-083801 and PEJ2018-003155, respectively. AR-J and MF are recipients of Postdoctoral Perfection Grants by MCIU (CP18/00111 and CD18/00091, respectively).

## Conflict of Interest

The authors declare that the research was conducted in the absence of any commercial or financial relationships that could be construed as a potential conflict of interest.

## Publisher's Note

All claims expressed in this article are solely those of the authors and do not necessarily represent those of their affiliated organizations, or those of the publisher, the editors and the reviewers. Any product that may be evaluated in this article, or claim that may be made by its manufacturer, is not guaranteed or endorsed by the publisher.
